# Study on Misalignment Angle Compensation during Scale Factor Matching for Two Pairs of Accelerometers in a Gravity Gradient Instrument

**DOI:** 10.3390/s18041247

**Published:** 2018-04-18

**Authors:** Xiangqing Huang, Zhongguang Deng, Yafei Xie, Ji Fan, Chenyuan Hu, Liangcheng Tu

**Affiliations:** 1 MOE Key Laboratory of Fundamental Physical Quantities Measurement & Hubei Key Laboratory of Gravitation and Quantum Physics, School of Physics, Huazhong University of Science and Technology, Wuhan 430074, China; hxq160@hust.edu.cn (X.H.); dzg_109@hust.edu.cn (Z.D.); xieyaphe@hust.edu.cn (Y.X.); fanji@hust.edu.cn (J.F.); 2 TianQin Research Center for Gravitational Physics and School of Physics and Astronomy, Sun Yat-sen University (Zhuhai Campus), Zhuhai 519082, China; 3 Institute of Geophysics, Huazhong University of Science and Technology, Wuhan 430074, China

**Keywords:** misalignment angle compensation, scale factor matching, magnetic force feedback accelerometer, gravity gradient instrument

## Abstract

A method for automatic compensation of misalignment angles during matching the scale factors of two pairs of the accelerometers in developing the rotating accelerometer gravity gradient instrument (GGI) is proposed and demonstrated in this paper. The purpose of automatic scale factor matching of the four accelerometers in GGI is to suppress the common mode acceleration of the moving-based platforms. However, taking the full model equation of the accelerometer into consideration, the other two orthogonal axes which is the pendulous axis and the output axis, will also sense the common mode acceleration and reduce the suppression performance. The coefficients from the two axes to the output are *δ_O_* and *δ_P_* respectively, called the misalignment angles. The angle *δ_O_*, coupling with the acceleration along the pendulous axis perpendicular to the rotational plane, will not be modulated by the rotation and gives little contribution to the scale factors matching. On the other hand, because of coupling with the acceleration along the centripetal direction in the rotating plane, the angle *δ_P_* would produce a component with 90 degrees phase delay relative to the scale factor component. Hence, the *δ_P_* component coincides exactly with the sensitive direction of the orthogonal accelerometers. To improve the common mode acceleration rejection, the misalignment angle *δ_P_* is compensated by injecting a trimming current, which is proportional to the output of an orthogonal accelerometer, into the torque coil of the accelerometer during the scale factor matching. The experimental results show that the common linear acceleration suppression achieved three orders after the scale factors balance and five orders after the misalignment angles compensation, which is almost down to the noise level of the used accelerometers of 1~2 × 10^−7^ g/√Hz (1 g ≈ 9.8 m/s^2^).

## 1. Introduction

Over the past two decades, there has been a continuing and growing interest in the ability of moving-based gravimeters and gravity gradiometers, and this attention is not only focused on the remarkable achievements that has been made, but also on the development of new methods and technologies in an attempt to find even better alternatives [1,2,3]. The conventional gravimeters record the intensity of the gravity field but are insensitive to the edges and shapes of targets. In contrast, gravity gradiometers measure the spatial rate of change in the gravity field, which captures the high frequency signal associated with near-surface lateral density variations, and directly reflects the edges and shapes of sources rather than just mass distribution. Thus the gravity gradiometer is thought to be 3-D gravity while the gravimeter is usually 1-D one [4,5]. Because of this characteristic, the gravity gradient measurement has been widely used in the fields of earth science, resource exploration, inertial navigation and so on [1,6,7,8,9]. Because the gravity field of the Earth is extremely weak, accompanied with a variety of noises from the measuring environment, it imposes extremely harsh requirements on gravity gradient measurement technology. Because of the well-known reasons, people often need to develop some new techniques and methods independently to realize gravity gradient measurement, ceaselessly searching advances in sensor systems, new methods, operational efficiency, data processing and interpretation, and so on [1,6,9,10,11,12,13,14,15,16,17].

The first technology to provide gravity gradient survey capability suitable for moving-base applications is the gravity gradiometer instrument (GGI) developed by Bell Aerospace (now Lockheed Martin) between 1975 and 1990′s [4,5,9]. The basic element of the GGI design consists of two pairs of accelerometers [9,10,11]. The four accelerometers are equi-spaced around the rotary stage with their sensitive axes tangential to the disk. The outputs of each pair of the accelerometers are summed to reject the in-plane common linear acceleration and double the gradient signal. The outputs of the two pairs of the accelerometers are subtracted to suppress the angular acceleration about the spin axis and double the gradient signal once again. GGI is intrinsically sensitive to the external acceleration because of the mismatch of the scale factors of accelerometers [10,11]. Unfortunately the consistency of the scale factors can hardly meet survey requirements according to the current state-of-art of manufacturing [9,10,11]. In addition, the balance of the scale factors is impossible to maintain for an extended period due to the material aging, temperature variance, and electronic component instabilities, etc. Therefore, automatically matching the scale factors is necessary for the common linear and angular acceleration rejection, so as to improve the dynamic performance of the GGI.

In our previous experiment of the scale factor matching [18], a small orthogonal signal at the spin frequency is found after the balance has been achieved. It will result in a residual sensitivity to the common linear acceleration. Further analysis shows that the orthogonal signal is produced by the misalignment angles of a pair of accelerometers [18]. The error contributions of the misalignment angles *δ_P_* and *δ_O_* are investigated during the scale factor matching. The angle *δ_O_*, coupling with the acceleration along the pendulous axis, would not be modulated by the rotation and contributes little to the scale factor matching. However, the angle *δ_P_*, coupling with the acceleration along the centripetal direction in plane, would produce an orthogonal component relative to the scale factors component. The *δ_P_* component is therefore in the same phase with the sensitive direction of the orthogonal accelerometers. Hence, the misalignment angle component can be compensated by injecting a trimming current proportional to the output of an orthogonal accelerometer into the torque coil.

Although the misalignment angles have a standard definition [19], the online misalignment angles compensation are discussed very little in the previous literature. To the best of our knowledge, the only publicly available information for misalignment compensation comes from the United States patent literature [20,21]. In order to reduce or compensate the unwanted noises from centripetal, misalignment, and nonlinear effects associated with GGI measurements, a sensor assembly, including an additional accelerometer and one or more rate sensor packages, is directly mounted on or within the GGI. It is claimed that an accelerometer was positioned at the center of the disk with its input axis parallel with the spin axis, and then this allowed for improved removal of errors due to nonlinearities and misalignments of the two pairs of the accelerometers. Besides, one or more angular rate sensors were mounted such that the input axes of the sensors were orthogonal to each other, and this could provide outputs to calculate the misalignment correction in real time or later in the data reduction process.

Here we report an alternative method to compensate the misalignment angles automatically during matching the scale factors of two pairs of the accelerometers in GGI. Compared with the method presented in [21], no extra sensors are needed during the compensation process. The experimental results show that the common linear acceleration is suppressed by three orders after scale factor matching and five orders after misalignment angle compensation, which almost reached the noise level of the used accelerometers of 1~2 × 10^−7^ g/√Hz.

## 2. Principle of the Misalignment Angle Compensation in Scale Factor Balance for a Pair of Accelerometers

### 2.1. The noise effect of the misalignment angle

**Figure 1:**
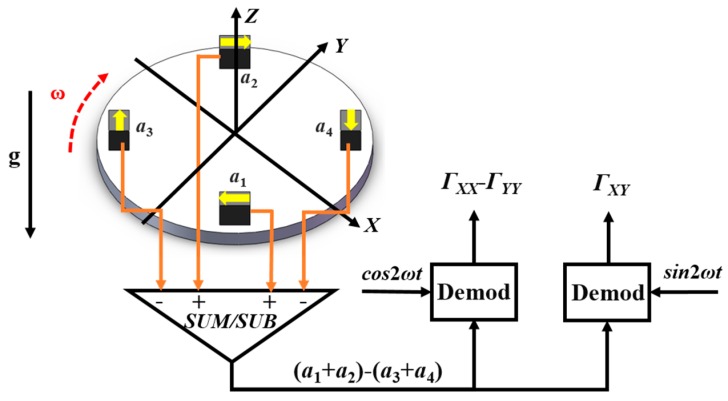
Schematic diagram of the rotating accelerometer GGI.

The schematic diagram of the rotating accelerometer GGI is shown in Figure 1. The GGI consists of two pairs of accelerometers. Each pair of accelerometers are set diametrically opposite to each other to suppress the in-plane common linear acceleration and to double the gradient signal. The two pairs of accelerometers are set mutually orthogonal to reject angular acceleration about the spin axis and double the gradient signal once again [9,10,11,20].

The output of each accelerometer is shown in Equation (1):

(1) $$a_{i}=-a_{COM}K_{Ii}\sin(\theta +\phi_{i})+\frac{1}{2}R(\Gamma_{XX}-\Gamma_{YY})\sin2(\theta +\phi_{i})+R\Gamma_{XY}\cos2(\theta +\phi_{i})$$

where *a*_i_, *K_Ii_*, *φ_i_* are the output, scale factor and position phase of the *i*th accelerometer respectively. The position phase of each accelerometer is *φ*_1_ = 0, *φ*_2_ = π, *φ*_3_ = π/2 and *φ*_4_ = 3π/2, respectively. *a_com_* is the in-plane common linear acceleration, *R* is the distance from the proof mass (PM) of the accelerometer to the center of the rotation and *Γ_XX_*− *Γ_YY_* and *Γ_XY_* are the gradient signals. The summed output of the four accelerometers is shown in Equation (2):

(2) $$\begin{array}{cc} \left(a_{1}+a_{2})-(a_{3}+a_{4}\right) & =-a_{COM}(K_{I1}-K_{I2})\sin\theta +a_{COM}(K_{I3}-K_{I4})\cos\theta \\ & +2R(\Gamma_{XX}-\Gamma_{YY})\sin2\omega t+4R\Gamma_{XY}\cos2\omega t \end{array}$$

The gradient signals *Γ_XX_*− *Γ_YY_* and *Γ_XY_* are modulated to the double spin frequency of the rotation, while the kinematic acceleration is modulated to the single spin frequency. Thus, the gradient signals and the noise are separated in the frequency domain. However, once the common linear acceleration contains a single spin frequency component, a disturbance to the double spin frequency component is unavoidable. Only when the scale factors of a pair of accelerometers are precisely matched (*K_I_*_1_ = *K_I_*_2_, *K_I_*_3_ = *K_I_*_4_), the noise effect of *a_com_* can be eliminated.

In our previous research, we have developed a scale factor adjustment method by trimming the feedback current of a magnetic force feedback accelerometer [22]. The PM is suspended with a thin cantilever spring, and its movement is sensed by the capacitive displacement transducer. A digital proportional integral differential (PID) controller is used to generate a current *I_f_* flowing through the torque coil mounted on the PM, which provides an equal force with an opposite direction to compensate the movement of the PM. In order to adjust the scale factor of the accelerometer, a trimming current *I_t_*, which is proportional to the feedback current by *It* = *p* × *If,* was injected into the torque coil. In this case, the scale factor of the accelerometer becomes *K_I_*= *K_I_*_0_/(1 + *p*), where *K_I_*_0_ is the original scale factor of the accelerometer. The adjustment method has been proven to be effective and easy to implement. The measurement range of the accelerometer is about 30 mg.

**Figure 2:**
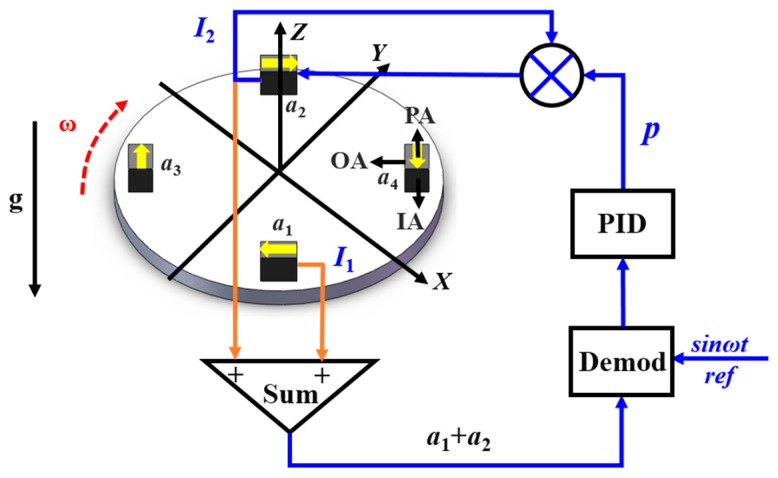
Schematic diagram of the scale factor balance loop of a pair of accelerometers.

Taking the pair of accelerometers *a*_1_ and *a*_2_ as example, the schematic diagram of the scale factor balance loop is depicted in Figure 2. The scale factor mismatch information is extracted by demodulating the summed output *a*_1_ + *a*_2_ with sin*ωt*. Subsequently, the PID controller can obtain a proper coefficient *p* for the scale factor balance. The output of *a*_2_ is multiplied by the coefficient *p* and injected into the torque coil, hence the scale factor is balanced [22].

However, after the balance has been achieved an orthogonal signal at the spin frequency is still observed, which results in a residual sensitivity to the common linear acceleration. For further analysis Equation (3) shows the complete model equation of an accelerometer [19]:

(3) $$\begin{array}{cc} a_{out}=I_{out}/K_{I} & =K_{0}+a_{I}-\delta_{P}a_{O}+\delta_{O}a_{P}\\ & +K_{II}a_{I}^{2}+K_{OO}a_{O}^{2}+K_{PP}a_{P}^{2}\\ & +K_{IO}a_{I}a_{O}+K_{IP}a_{I}a_{P}+K_{OP}a_{O}a_{P}+\ldots \end{array}$$

where *a_out_* is the indicated acceleration, *I_out_* is the output current of the accelerometer, *K_I_* is the scale factor, and *K*_0_ is the bias. *a_I_*, *a_O_* and *a_P_* are the applied acceleration components along the true input (sensitive) axis IA, output axis OA, and pendulous axis PA, respectively. The three axes are mutually orthogonal. *δ_P_* and *δ_O_* are the misalignment angles of the input axis with respect to the input reference axis about the pendulous axis and the output axis, respectively. *K_II_*, *K_OO_* and *K_PP_* are second order coefficients. *K_IO_*, *K_IP_* and *K_OP_* are the cross-coupling coefficients. All these coefficients can be measured with a centrifuge [23]. The second order coefficients and cross-coupling coefficients, which will generate higher order harmonic signals, have been discussed in our previous work [18]. By taking the first order coefficients into consideration, the output of the four accelerometers can be expressed as:

(4) $$I_{i}=a_{COM}(-K_{Ii}\sin(\omega t+\varphi_{i})+K_{Ii}\delta_{Pi}\cos(\omega t+\varphi_{i}))+K_{Ii}\delta_{Oi}a_{V}$$

where *I_i_*, *K_Ii_, δ_Pi_*, *δ_Oi_*, *φ_i_* (*I* = 1, 2, 3, 4) are the output, scale factor, two misalignment angles and position phase of the *i*th accelerometer, respectively. The position phases of the four accelerometers are *φ*_1_ = 0, *φ*_2_ = π, *φ*_3_ = π/2 and *φ*_4_ = 3π/2. *ω* is the spin frequency. The in-plane common acceleration *a_COM_* is modulated to the spin frequency of the disk. *a_V_* is the vertical acceleration perpendicular to the plane, hence is not modulated by the rotation and contributes little to the scale factor balance. However, once the common acceleration *a_COM_* contains a single frequency component, such as *a_COM_*= *A_s_*sin*ωt + A_c_*cos*ωt*, a noise component contributing to the double frequency where the gradient signal stays is unavoidable given by:

(5) $$\begin{array}{cc} I_{1}+I_{2} & =\frac{A_{c}(K_{I1}\delta_{P1}-K_{I2}\delta_{P2})-A_{s}(-K_{I1}+K_{I2})}{2}\cos2\omega t\\ & +\frac{A_{c}(K_{I1}\delta_{P1}-K_{I2}\delta_{P2})+A_{s}(-K_{I1}+K_{I2})}{2}\sin2\omega t \end{array}$$

Using Equation (5), we find the misalignment angle *δ_P_* would contribute a noise to the gradient signal extraction the same as scale factor mismatch. Therefore, the misalignment angle also needs compensation during the scale factor balance to suppress the common linear acceleration. In an actual survey environment, the scale factor and misalignment angle may change with temperature, material aging, structure creep, and stress release, etc. Thus, the scale factor balance and misalignment angle compensation are required to be online and automatic.

### 2.2. The principle of the misalignment angle compensation

As shown in Equation (4), the scale factor mismatch information of *a*_1_ and *a*_2_ is modulated by sin*ωt* while the misalignment angle information is modulated by an orthogonal signal cos*ωt.* Therefore, the scale factor mismatch and misalignment angle information can be distinguished by demodulation with sin*ωt* and cos*ωt*, respectively. As shown in Equation (3), the misalignment angle component of the summed output *a*_1_ + *a*_2_ is in the same phase with the scale factor component of an orthogonal accelerometer *a*_4_. Inspired by the scale factor adjustment method, we figure out that the misalignment angle can be compensated by trimming the feedback current with the output of orthogonal accelerometer. 

**Figure 3:**
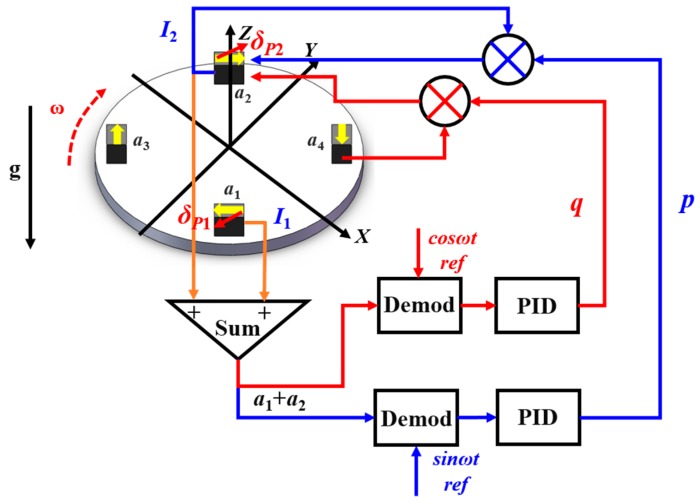
Schematic diagram of the scale factor balance loop and the misalignment angle compensation loop for a pair of accelerometers. The blue signal flow is for scale factor balance and the red signal flow is for misalignment angle compensation.

For example, the misalignment angle of the pair of accelerometers (*a*_1_ and *a*_2_) can be compensated by injecting the output of an orthogonal accelerometer (*a*_4_) into the torque coil of accelerometer (*a*_2_). The active feedback loops for matching the scale factors and compensating the misalignment angles are schematically depicted in Figure 3.

After demodulation a group of proper coefficients *p* and *q* are calculated out by two PID-controllers, respectively. The output of the accelerometer *a*_2_ is multiplied by *p* and *a*_4_ is multiplied by *q*, and then are injected into the torque coil of *a*_2_ for scale factor balance and misalignment angle compensation. The summed output after the balance and compensation is given as:

(6) $$\begin{array}{cc} {I^{\prime }}_{1}+{I^{\prime }}_{2} & =a_{COM}(-K_{I1}+\frac{K_{I2}}{1-p}+\frac{qK_{I4}\delta_{P4}}{1-p})\sin\omega t\\ & +a_{COM}(K_{I1}\delta_{P1}-\frac{K_{I2}\delta_{P2}}{1-p}+\frac{qK_{I4}}{1-p})\cos\omega t \end{array}$$

As Equation (6) shows, the summed output after balance and compensation consists of two components. One is the scale factor balance component (sin*ωt*), and the other is the misalignment angle compensation component (cos*ωt*). In our previous experiment, only scale factor balance is taken into consideration with *q* = 0 in Equation (6), hence an orthogonal component coupled with the misalignment angles cannot be eliminated. A residual sensitivity to the common linear acceleration is therefore unavoidable. To suppress the common linear acceleration, the magnitudes of these two components should be adjusted to zero. Normally the misalignment angle of the accelerometer is of the order of 10^−2^~10^−3^ rad, which contributes much little than the scale factor. Thus, from Equation (6), we can find out that the coefficient *p* is the main contributor to the scale factor balance, while coefficient *q* contributes the most to the misalignment angle compensation. The scale factor balance coefficient *p* and misalignment angle compensation coefficient *q* can be solved using the following equation:

(7) $$\left\{ \begin{array}{l} p=\frac{K_{I1}-K_{I2}+(K_{I1}\delta_{P1}-K_{I2}\delta_{P2})\delta_{P4}}{K_{I1}(1+\delta_{P1}\delta_{P4})}\\ q=-\frac{K_{I2}(\delta_{P1}-\delta_{P2})}{K_{I4}(1+\delta_{P1}\delta_{P4})} \end{array}\right.$$

which verified the feasibility of the balance and compensation method. The misalignment angles of the other pair of accelerometers *a*_3_ and *a*_4_ can be compensated by similar method. The misalignment information can be extracted by demodulating with cos*ωt* and compensated with an orthogonal accelerometer *a*_1_ or *a*_2_.

## 3. Experimental Results

**Figure 4:**
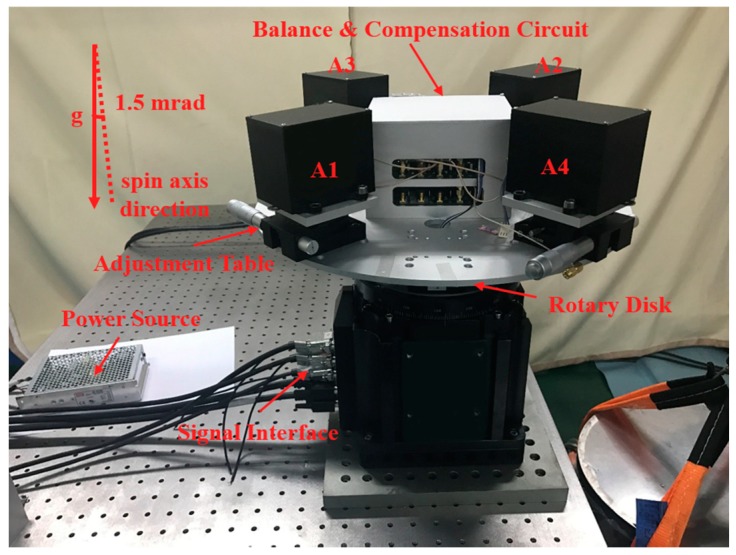
Experimental setup for the GGI.

The experimental setup is shown in Figure 4. The GGI is mounted on a commercial rotary stage and the rotating frequency is set to 0.125 Hz. The base line from the PM to the center of the rotary disc is about 135 mm. To reveal the mismatch of the scale factors of a pair of accelerometers, the spin axis is purposely inclined from the vertical axis with an angle of 1.5 mrad. The reference demodulation signals sin*ωt* and cos*ωt* are directly read out from the grating encoder integrated in the rotary stage online. The balance and compensation circuit consists of 20-bit analog-to-digital converters, a field programmable gate array (FPGA) and 20-bit digital-to-analog converters. All the control algorithms are implemented in the FPGA. Therefore, no extra compensation circuit added is needed for the misalignment angles compensation loops.

**Figure 5:**
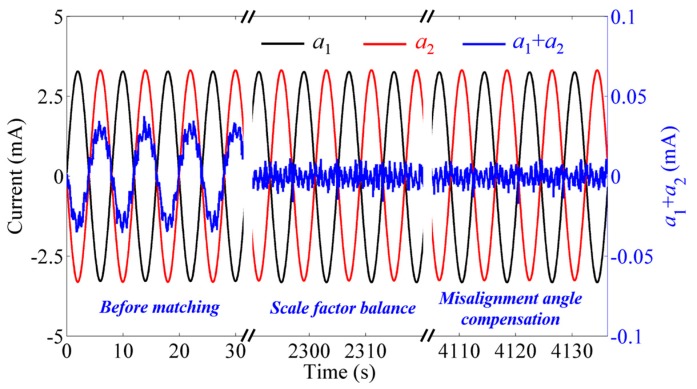
Current output of a pair of accelerometers *a*_1_, *a*_2_ and the summed output *a*_1_ + *a*_2_.

After the disk is working stably, the scale factor balance loop waits for a sufficient period of time until the mismatch information of a pair of accelerometers is filtered out. The misalignment angle compensation loop then starts to work after the scale factor balance has been achieved. In fact, the balance loops and the compensation loops work continuously and accurately. The outputs of a pair of accelerometers *a*_1_, *a*_2_ and the summed output *a*_1_ + *a*_2_ is shown in Figure 5. At the beginning, the magnitudes of *a*_1_ and *a*_2_ are different due to the mismatch of the scale factor. The summed output *a*_1_ + *a*_2_ indicates an obvious periodic signal at the spin frequency. The outputs of *a*_1_ and *a*_2_ eventually become equal in amplitude and the spin frequency component in the summed output hence disappears, indicating that the balance and compensation have been completed.

To clearly figure out the effect of the scale factor balance and misalignment angle compensation, the sin*ωt* and cos*ωt* components of the outputs and the summed output of the pair of accelerometers are obtained by least square fitting, and are listed in Table 1.

Before matching, the magnitude of the sin*ωt* and cos*ωt* components of the two accelerometers are different. After scale factor balance, the magnitude of the sin*ωt* component of the two accelerometers are equal to each other. Finally, after misalignment angle compensation, the magnitudes of the cos*ωt* component of the two accelerometers are also identical to each other. Consequently, the spin frequency in the summed output disappears.

**Figure 6:**
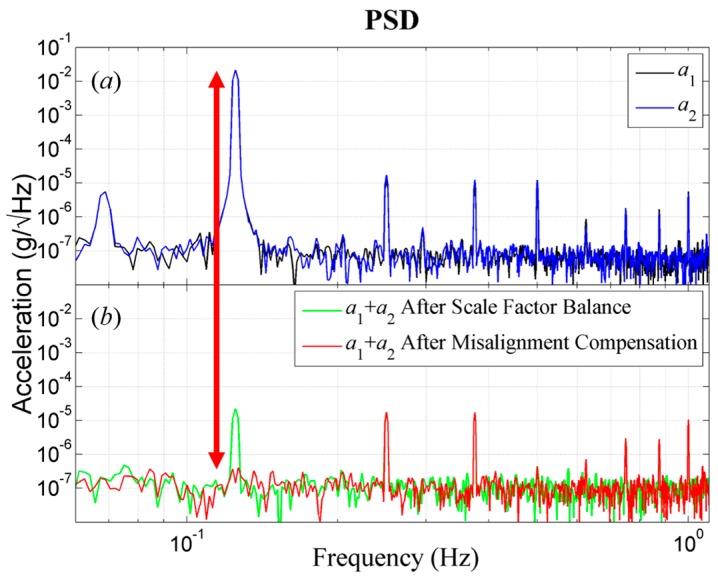
The acceleration PSD of the pair of accelerometers *a*_1_, *a*_2_ and *a*_1_ + *a*_2_ after scale factor balance and after misalignment angle compensation. (**a**) The PSD of accelerometer *a*_1_ and *a*_2_ before scale factor balance. (**b**) The PSD of *a*_1_ + *a*_2_ after scale factor balance and after misalignment angle compensation.

In the frequency domain, the acceleration power spectral density (PSD) curves of *a*_1_, *a*_2_ and *a*_1_ + *a*_2_ after scale factor balance and after misalignment angle compensation are shown in Figure 6.

The PSD curves of accelerometers *a*_1_ and *a*_2_ are shown in Figure 6a before the scale factor balance and misalignment angle compensation. The noise levels of the used accelerometers are about 1~2 × 10^−7^ g/√Hz. The spin frequency component of the combined output of *a*_1_ and *a*_2_ reveals the residual sensitivities to common linear acceleration. The imperfect movement of the rotary stage, such as additional angular acceleration, radially twisting movement and the brush of the motor also introduce higher harmonic responding components. The PSD curves of combined output are shown in Figure 6b (green) after scale factor balance. The common linear acceleration achieves three orders of suppression. After misalignment angle compensation the common linear acceleration finally achieves five orders of suppression and reaches the noise level of the accelerometers (Figure 6b, red). The PSD results further validate the effectiveness of the misalignment compensation method during the scale factor balance. The other pair of accelerometers *a*_3_ and *a*_4_ performed almost the same.

## 4. Conclusions and Prospects

This paper presents the influence of the misalignment angle and the alternative online compensation method for the in-plane common acceleration suppression in the GGI with the output of an orthogonal accelerometer. Analysis shows that the misalignment angle can lead to errors in the gradient signal extraction as well as the scale factor mismatch. Thus, the misalignment angle compensation technology is necessary. Using the same circuit of scale factor adjustment, we develop a method to compensate the misalignment angle by injecting the output of an orthogonal accelerometer into the torque coil. Experimental results show that the first order common linear acceleration has been suppressed by five orders, reaching the noise level of the used accelerometers. No additional sensors or circuit are needed for the misalignment angle compensation and that makes it simple for implementation.

However, to extract a meaningful gradient from GGI, a number of technological improvements are still needed. First of all, the major limitation of suppression is the noise level of the used accelerometer. Besides, each accelerometer unavoidably includes higher order nonlinear and cross-coupling components because of the imperfection of the material and mismatch of assembling. Additionally, non-ideal movement of the rotary stage will induce many harmonic components. All these harmonic components may degrade the quality of the gravity gradient signals.

To further lower the noise level of the accelerometer is a challenging but imminent objective, and the good news is we have some options [24]. Precision control of the rotary stage is also taken into consideration. In addition, the technique to reduce or null the even order and cross-coupling error coefficients is being developed to further improve the sensitivity of the GGI.

## Figures and Tables

**Table 1:** The sin*ωt* and cos*ωt* components of *a*_1_, *a*_2_ and *a*_1_ + *a*_2_ before matching, after scale factor balance and after misalignment angle compensation.

Output Current (mA)	Before Matching		After Scale Factor Balance		After Misalignment Angle Compensation	
	sin*ωt*	cos*ωt*	sin*ωt*	cos*ωt*	sin*ωt*	cos*ωt*
*a*_1_	3.3830(3)	−0.0747(3)	3.3953(3)	−0.0763(3)	3.3963(3)	−0.0060(3)
*a*_2_	−3.4150(3)	0.0753(3)	−3.3957(3)	0.0750(3)	−3.3963(3)	0.0060(3)
*a*_1_ + *a*_2_	−0.0322(2)	0.0006(2)	−0.0001(2)	−0.0013(2)	−0.0000(2)	−0.0000(2)
